# Dietary Effect of Curcumin on Amino Acid, Fatty Acid, and Volatile Compound Profiles of Chicken Meat

**DOI:** 10.3390/foods13142230

**Published:** 2024-07-16

**Authors:** Ying Shu, Fengyang Wu, Wei Yang, Wenhui Qi, Runyang Li, Zhisheng Zhang

**Affiliations:** 1 College of Food Science and Technology, Hebei Agricultural University, Lekai South Avenue, Baoding 071000, China; hebaushuying@hebau.edu.cn (Y.S.); fengyangwu2020@163.com (F.W.); wenhui406@yeah.net (W.Q.); runyangli@163.com (R.L.); 2 Hebei Layer Industry Technology Research Institute, Economic Development Zone, Handan 545000, China; 3 Institute of Animal Husbandry and Veterinary Medicine of Hebei Province, Dongguan Avenue, Baoding 071030, China; realyangvv@163.com

**Keywords:** curcumin, meat, amino acid, fatty acid, volatile compounds

## Abstract

This study investigated the dietary effect of curcumin (CUR) on amino acid, 5′-nucleotides, fatty acid, and volatile compound profiles of chicken meat. A total of 400 healthy 1-day-old broiler male chicks were divided into 4 groups (*n* = 10) and fed either a basal diet or a diet with the addition of CUR with concentrations of 100 mg/kg, 150 mg/kg, and 200 mg/kg for 43 days. The results show that the addition of CUR in chicken diets is conducive to promoting the deposition of amino acids and increasing the content of 5′-nucleotides in chicken meat, reducing the contents of saturated fatty acid (SFA) and C20:4 n6 but increasing the ratio between polyunsaturated fatty acid (PUFA) and SFA. In addition, the volatile compound profile shows that the main volatile compounds in chicken meat are aldehydes (including hexanal, heptanal, octanal, and nonanal), with significant increases in their contents observed among chickens in the CUR-intake group. Moreover, it has been found that (E, E)-2,4-nonadienal, trans-2-decenal, benzaldehyde, and trans-2-octenal in chicken meat can significantly increase its overall aroma, and the addition of CUR with 150 mg/kg had the best effect on improving nutritional quality and flavor of chicken meat. This study provides a basis for the comprehensive utilization of CUR as a feed additive with the potential to substitute antibiotics.

## 1. Introduction

Poultry meat is viewed as one of the largest sources of animal protein in people’s diets around the world [1]. With the advantages of high-quality nutritional components, low cholesterol content, diverse amino acid compositions, and relatively high digestibility, it has been greatly favored by consumers. Antibiotics have been widely used in the poultry industry for disease prevention and treatment as well as production promotion [2,3]. Nevertheless, the overuse of antibiotics has led to various adverse consequences, such as drug residues, bacterial resistance, and environmental pollution. In 2006, 2017, and 2020, Europe, the United States, and China banned the addition of antibiotics in poultry and livestock feed in succession [4,5]. Now, natural plant extracts rich in phenolic compounds have been widely studied because of their good effects in delaying the oxidation of fats and proteins, enhancing antioxidation, improving growth performance, and improving quality [6,7].

Curcumin (CUR) is a hydrophobic phenolic compound extracted from the rhizomes of *Curcuma longa* L. [8]. This compound exhibits a wide range of pharmacological effects, including anti-inflammation [9,10], anti-oxidation [11,12], anti-tumor [13,14], anti-bacteria [15,16], anti-virus [17], digestion promotion [18], and immune regulation [19,20]. In recent years, some studies have been performed on the effects of CUR in the poultry farming industry. These studies showed that the use of CUR as a feed additive to chicken diets can affect the growth performance of poultry and various quality indices of poultry meat, improving the feed conversion efficiency, effectively reducing the deposition of abdominal and liver fats, and consequently improving meat quality [21,22,23,24]. The addition of CUR in the diets of Cherry Valley ducks improved their muscle quality and enhance their antioxidant activities [25]. In addition, the addition of CUR and acetylsalicylic acids in diets of heat-stressed broiler chickens significantly reduced the content of MDA in their breast muscle tissue, improving their antioxidant capacities [26]. The results of the previous experiment of our team showed that adding CUR to the diet of broilers could reduce the content of MDA and improve the processing quality and antioxidant activity of chicken meat [27], so we hypothesized that the use of CUR as a feed additive could affect nutritional components and flavor by influencing the antioxidant capacity of chicken meat. Therefore, a comprehensive evaluation was conducted in this study on the dietary effects of CUR on amino acid, 5′-nucleotides, fatty acid, and volatile compound profiles of chicken meat.

## 2. Materials and Methods

### 2.1. Experimental Design, Animal, and Diet

This research was approved by the Ethics Committee of Hebei Agricultural University (Number: 2022003). All animal experiments that comply with the REACH guidelines were conducted in accordance with the British Animals (Scientific Procedures) Act 1986 and its relevant guidelines, as well as the EU Directive 2010/63/EU on the protection of animals used for scientific purposes. The CUR (with a purity of ≥95%) used in this research was purchased from Chenguang Biotech Group Co., Ltd., Handan, China. The basal ration of a corn–soybean meal-type ration was prepared according to the Feeding Standard of Chickens (NY/T 33-2004) in China [28]. Its composition and nutritional levels are listed in Table 1. A total of 400 healthy 1-day-old Arbor Acres broiler male chickens with similar body weights (44.18 ± 1.05 g) were selected and randomly divided into four groups, with 10 replicates in each group and 10 chicks in each replicate. Chicks were reared on soft bedding cages and housed in a well-ventilated room. Artificial light was provided for 12 h until the end of experiment. Heaters were installed in the experimental chicken room to regulate the environmental temperature according to body requirement of the chicks. An experimental period of 43 days was applied in this research. Chickens in the control group (C0) were fed with a basal diet, while chickens in the CUR-addition group were individually fed with basal diets with the addition of curcumin with concentrations of 100 mg/kg (C1), 150 mg/kg (C2), and 200 mg/kg (C3). During the experiment, test subjects could freely eat, feed, and drink water.

### 2.2. Sample Collection

At the end of the experiment, one broiler chicken with a body weight close to the average level was selected in each replicate (a total of eight chickens in each group). After a 12-h period of no feeding, all broiler chickens that were selected underwent euthanasia through cervical dislocation. Left breast meat samples were collected from among these chickens. With the removal of extra fats, these meat samples were divided into small pieces and frozen in liquid nitrogen and vacuum-packed directly, then stored at −80 °C for further measurement.

### 2.3. Amino Acid Measurement

The content of amino acids in the sample meat was measured using high-performance liquid chromatography (HPLC, Waters e2695, Waters Corporation, Milford, MA, USA). A sample with a weight of 1 g was precisely weighed and then adjusted to volume after a 24-h hydrolysis period under 110 °C in 6 mol/L hydrochloric acid, followed by filtration and evaporation. The content of amino acids was measured through the derivation of 2,4-dinitrochlorobenzene. The corresponding mobile phases A and B were a 0.25% anhydrous sodium acetate solution and chromatography-grade acetonitrile, respectively. 

The chromatographic conditions applied in the experiment are as follows: column temperature = 43 °C; detection wavelength = 360 nm; and mobile-phase flow rate = 1 mL/min. For the gradient elution program, during the period of 0–10 min, the volume ratio between mobile phases A and B was set at 82:18. During the period of 10–15 min, the volume ratio between mobile phases A and B was set at 71:29. During the period of 15–25 min, the volume ratio between mobile phases A and B was set at 66:34. During the period of 25–30 min, the volume ratio between mobile phases A and B was set at 45:55. During the period of 30–37 min, the volume ratio between mobile phases A and B was set at 40:60. During the period of 37–45 min, the volume ratio between mobile phases A and B was set at 82:18.

### 2.4. 5′-Nucleotide Measurement

A meat sample with a weight of 2.5 g was weighed and added into a centrifuge tube. The sample was homogenized using 20 mL of 5% HClO_4_ and was then centrifuged at 12,000 rpm for 20 min. After collection of the supernatant, the precipitate was homogenized using a 5% HClO_4_ solution and then centrifuged. After a combination of the secondary supernatant, the pH value of the solution was adjusted to 6.5, with its volume adjusted to 50 mL. Subsequently, a 0.22 μm filtration was conducted on the solution for further analysis. The filtrate was analyzed using HPLC (Waters e2695, Waters Corporation, USA) combined with a UV detector (254 nm) [29,30].

The analysis was conducted with the following conditions: chromatographic column = XBridge-C18-T (5 μm, 4.6 mm × 250 mm); column temperature = 25 °C; mobile phase A = phosphate saline buffer (1.40 mmol/L C_16_H_37_NO_4_S; 0.01 mol/L K_2_HPO_4_; pH 3.2); mobile phase B = methanol; flow rate = 1 mL/min; injection volume = 10 μL; isocratic elution; A:B = 98:2, 25 min.

### 2.5. Fatty Acid Measurement

An NaOH-methanol solution (2%) with a volume of 8 mL was added to the extracted sample, which then underwent a water bath at 80 °C for 20 min. Subsequently, a 15% boron trifluoride-methanol solution with a volume of 7 mL was added to the sample, which then underwent a water bath at 80 °C for 2 min. After the sample cooled down, an n-heptane solution with a volume of 10 mL was added to the sample and mixed well. Anhydrous sodium sulfate with a weight of 2 g was added to the sample for water absorption. Subsequently, gas chromatography was performed. Sample separation and quantification were conducted using a gas chromatograph-mass spectrometer (GCMS-QP2020 NX, Shimadzu Corporation, Kyoto, Japan) with the following conditions. Chromatographic column = SH-RXI-5SIL MS (30 m × 0.25 μm, 0.25 μm, Shimadzu Corporation, Japan); carrier gas = helium; flow rate = 1.0 mL/min; injection volume = 1.0 μL; injection port temperature = 270 °C; and injection method = splitless. The initial temperature of the column heater was set at 100 °C and maintained for 13 min. The temperature was then increased to 180 °C at a rate of 10 °C/min and was held there for 20 min. Subsequently, it was increased to 200 °C at a rate of 1 °C/min and was held there for 2 min. Lastly, the temperature was increased to 230 °C at a rate of 4 °C/min, and was held there for 12.5 min. Under 70 eV, quantitative and qualitative analyses of fatty acids were conducted using an external standard method with ion source and interface temperatures set at 250 °C and 280 °C, respectively [31].

### 2.6. Analysis of Volatile Flavor Components Using SPME-GC-MS

According to the literature [32], a sample with a weight of 2.00 g was precisely weighed and added into a 20 mL headspace vial. After that, 1.0 μL of 2-methyl-3-heptanone solution (0.408 μg/μL) was added into the vial, which was then sealed. The vial underwent a 55 °C water-bath equilibrium for 20 min. Subsequently, volatile components were adsorbed using a 75 μm CAR/PDMS fiber (Supelco, Inc., Bellefonte, PA, USA) at 55 °C for 40 min. After the completion of the adsorption, a 5-min desorption at 250 °C was immediately performed on the fiber. GC-MS analysis was conducted using a GCMS-QP2020 NX gas chromatograph-mass spectrometer (Shimadzu Corporation, Japan) with the following conditions: chromatographic column = SH-RXI-5SIL MS (30 m × 0.25 μm, 0.25 μm, Shimadzu Corporation, Japan); carrier gas = helium (≥99.999%); flow rate = 1.0 mL/min (constant); and injection port temperature = 250 °C (splitless). The initial temperature was set at 40 °C and maintained for 5 min. The temperature was then increased to 100 °C at a rate of 15 °C/min. After that, it was further increased to 220 ℃ at a rate of 5 °C/min and was held there for 2 min. Lastly, the temperature was increased to 260 °C and was held there for 5 min. Under 70 eV, electron impact mass spectrometry was performed with an ion-source temperature of 230 °C and an *m*/*z* scanning range of 40–450 μm. The obtained mass spectra were compared with the US National Institute of Standards and Technology (NIST) Mass Spectral Library (version 17.0) database to identify the volatile components. The gas chromatographic peak area of the volatile component was compared with the internal standard area to determine the concentration of the volatile component. Odor activity values (OAVs) were calculated using the following formula to determine the odor-active compounds:

(1) $${OAV}_{i}=\frac{C_{i}}{{OT}_{i}}$$

where *C_i_* represents the concentration of the volatile compound, and *OT_i_* represents the odor threshold in water.

### 2.7. Statistical Analysis

The experimental results are expressed as the mean ± standard error (SE). The difference significance test was conducted using an ANOVA in SPSS23.0. Duncan’s method was used in multiple comparisons, with a *p*-value lower than 0.05 (*p* < 0.05) indicating a significant difference. 

## 3. Results and Discussion

### 3.1. Analysis of Amino Acid Content

Amino acids are an important precursor substance of meat flavor compounds and a major indicator for assessing the quality of meat flavor [33]. They play a significant role in the formation of flavor compounds in chicken meat [34]. In this study, it was found that the addition of CUR with different concentrations in the diets of chickens presented different influences on the contents of amino acids in chicken meat (see Table 2). Compared to the chicken meat in the C0 group, the meat in the C2 group exhibited the highest concentrations of essential amino acids (EAA) and non-essential amino acids (NEAA) (*p* < 0.05). Except for glycine (Gly), chicken meat in the C2 group presented the highest contents of amino acids (*p* < 0.05). In addition, chicken meat in the C0 group exhibited the highest content of Gly (*p* < 0.05), while chicken meat in the C2 group presented the highest content of serine (Ser) (*p* < 0.05).

EAA is an essential substance for the human body. However, it cannot be produced by the human body and must be obtained through food. Food with a higher EAA content exhibits a higher nutritional value [35]. At the same time, the contents of certain types of amino acids are closely associated with the normal metabolism of the human body. For instance, threonine (Thr) can promote muscle growth and facilitate fat metabolism. Leucine (Leu) can induce enhanced activities of antioxidases, improving the antioxidant capacity of the human body. Serine, as a metabolic intermediate, participates in the synthesis of methionine (Met), glycine (Gly), and cysteine (Cys), primarily playing a role in lipid metabolism, the immune system, and the central nervous system. Although Ser is not an essential amino acid, it plays a crucial role in a series of metabolic processes and the maintenance of bodily functions. The reason why chicken meat in the C2 group presents an increased Ser content could lie in that chickens fed with rations with the addition of CUR exhibit increased intake and turnover amounts of proteins. In addition, Gly can be directly converted into Ser. Therefore, the reason why there is a decreased Gly content could lie in the fact that Gly is converted into Ser through the catalysis of serine hydroxymethyltransferase.

The content of amino acids in muscle has a significant influence on the nutritional value of chicken meat. Moreover, some amino acids are key flavor compounds in chicken meat. Amino acids can react with reducing sugars, triggering the Maillard reaction, which constitutes one of the primary ways to form meat flavor [34]. In addition, amino acids can react with α-dicarbonyl compounds, triggering the Strecker degradation reaction, the product of which, Strecker aldehyde, is a primary source of meat flavor. Glutamate (Glu) and aspartic acid (Asp) are important flavor amino acids. During the hydrolysis process of the protein–peptide chain, these two amino acids can bind with sodium ions, leading to the formation of an umami taste [36,37]. Compared with the chicken meat in the C0 group, the chicken meat in the C2 group presented the highest contents of BAA, SAA, and FAA (*p* < 0.05). Sweet-tasting amino acids in chicken meat primarily include threonine (Thr), lysine (Lys), and serine (Ser). On the other hand, bitter-tasting amino acids in chicken meat are mostly important flavor enhancers involved in the Maillard reaction during the formation process of flavor. This study shows that bitter-tasting amino acids in chicken meat primarily include valine (Val), isoleucine (Ile), and methionine (Met). The results indicate that chickens fed with rations with the addition of CUR present a better overall flavor in their meat. In addition, it has been found in this research that chickens fed with rations with the addition of CUR exhibit EAA/TAA and EAA/NEAA ratios in their meat, which are close to those ideal ratios recommended by FAO/WHO. Changes in EAA, FAA, and TAA contents in chicken meat after the addition of CUR in chicken diets were investigated in this study. It indicated that chicken meat in the C2 group presented the most beneficial addition amount of CUR to the deposition of AA, and this amount can facilitate an improvement in the nutritional value and the formation of flavor in chicken meat [38].

### 3.2. Nucleotide Analysis

Flavor 5′-nucleotides include GMP, AMP, and IMP. These 5′-nucleotides and flavor amino acids can present a coordination effect of umami enhancement [39,40]. The effect data of different addition amounts of CUR on the nucleotide content in chicken meat are listed in Table 3. From this table, it can be seen that chickens fed with rations with the addition of CUR present significantly higher contents of 5′-nucleotides in their meat than those of chicken meat in the C0 group (*p* < 0.05). Specifically, chicken meat in the C2 group exhibited the highest contents of GMP, which were significantly higher than those of chicken meat in all other groups (*p* < 0.05). In addition, chicken meat in the C1 group presented the highest contents of AMP and IMP, which were significantly higher than those of chicken meat in all other groups (*p* < 0.05). The results show that the addition of CUR in chicken diets can significantly increase the contents of 5′-nucleotides in chicken meat, exhibiting an improvement effect on the contents of flavor 5′-nucleotides in chicken meat. This indicates that the addition of CUR in chicken diets can improve the contents of 5′-nucleotides in chicken meat, improving its flavor. This may be related to the increased level of IMP or GMP, which results from the improved antioxidant capacity of chicken meat [41].

### 3.3. Fatty Acid Analysis

From the perspective of human health, chicken meat can be regarded as a high-quality resource of essential fatty acids and proteins for the human body [42]. The tenderness, flavor, and nutritional value of chicken meat are all affected by the composition of fatty acids, which is greatly affected by the dietary control of chickens. The effect data of the addition of CUR in chicken diets on the contents of fatty acids in chicken meat are listed in Table 4. From the table, it can be seen that primary fatty acids detected in chicken meat included C16:0, C18:1 n-9 c, C18:2n-6, and C20:4n6.

The addition of CUR in chicken diets exerts a certain impact on the total SFA contents in chicken meat. SFAs in chicken meat are mostly palmitic acids (C16:0) and stearic acids (C18:0), indicating that these two fatty acids play important roles in the formation of chicken meat flavor. Compared with the C0 group, different experimental groups presented significant differences in their contents of palmitic acids (C16:0) (*p* < 0.05). Chicken meat in the C3 group presented the lowest contents of palmitic acids, which were significantly lower than those of chicken meat in the C0 group (*p* < 0.05). After the addition of CUR in chicken diets, chicken meat in the C2 group presented reduced contents of SFA fatty acids, with more significantly reduced contents of SFA fatty acids among chicken meat in the C3 group. This indicates that an appropriate CUR addition amount can decrease the SFA content in chicken meat. It is worth noting that among different dosage groups, chicken meat in the C1 group presented significantly increased stearic acid (C18:0) and total SFA contents with decreased concentrations of oleic acids (C18:1n-9c) and monounsaturated fatty acid (MUFA). Analysis shows that the possible reason is that the addition of CUR in chicken diets leads to a reduced conversion efficiency of stearic acid (C18:0) to oleic acid (C18:1n-9c). However, its mechanism is not fully understood [43].

MUFAs in chicken meat primarily include oleic acids (C18:1n9c) and palmitoleic acids (C16:1), while PUFAs in chicken meat primarily include linoleic acids (C18:2n6c) and arachidonic acids (C20:4n6). Among all MUFAs, chicken meat in the C0 group presented the highest contents of oleic acids, which were significantly higher than those of chicken meat in the C1 and C2 groups (*p* < 0.05). Chicken meat in the C1 group exhibited the highest contents of C14:1, C16:1, and C20:1, which were significantly higher than those of chicken meat in all other groups (*p* < 0.05). The contents of C20:4n6 in chicken meat in the C1 group were not significantly different from those of chicken meat in the C0 group but significantly lower than those of chicken meat in the C2 and C3 groups (*p* < 0.05). Meanwhile, chicken meat in the C2 group presented the highest contents of C20:4n6 (AA), which were significantly higher than those of chicken meat in all other treatment groups (*p* < 0.05). Chicken meat in the C2 group exhibited the lowest contents of C14:1, C16:1, and C18:1n9c. Among them, the contents of C14:1 in chicken meat in the C2 group were significantly lower than those of chicken meat in the C1 and C3 groups (*p* < 0.05), but not significantly different from those of chicken meat in the C0 group. The contents of C16:1 and C18:1n9c in chicken meat in the C2 group were significantly lower than those of chicken meat in the C0, C1, and C3 groups. PUFA is an essential substance for the human body, and a certain intake of PUFA has many benefits for human health [42]. A relatively high intake of n-3PUFA through diet is conducive to controlling cardiovascular diseases among humans. Therefore, an increase in the content of n-3PUFA can more specifically reflect the improvement in the food health indicators [43,44]. n-6 PUFA has the effects of maintaining the blood–lipid balance and preventing cardiovascular diseases. The study results showed that chicken meat in the C2 group exhibited the highest contents of C20:5n3 (EPA), C20:4n6 (AA), C22:6n-3 (DHA), and total PUFA (*p* < 0.05), indicating the potential of CUR for enriching PUFA in chicken meat [39]. Chicken meat with a higher PUFA/SFA ratio presents better quality. The results indicate that an appropriate addition amount of CUR in rations of broiler chickens can improve the nutritional value of chicken meat by impacting the fatty-acid composition of chicken breast meat. The reason could lie in the fact that antioxidant components in CUR can prevent the oxidation of PUFA, thus reducing the oxidation of fatty acids in chicken breast meat and consequently improving the quality of chicken meat. Similar effects have also been reported for other additives with antioxidant activity [43,45].

### 3.4. Analysis of Volatile Compounds

It can be seen that a total of 78 volatile compounds were detected among all chicken breast meat samples (Table 5). These compounds can be divided into 7 chemical classes, including 21 aldehydes, 19 hydrocarbons, 12 ketones, 7 alcohols, 4 esters, 10 aromatic compounds, 3 acids, and 2 heterocyclic compounds. Meanwhile, changes in varieties and contents of flavor compounds in chicken meat can be observed after the addition of CUR in chicken diets.

Meat flavor is a key factor for consumers when judging the acceptability of meat. During the cooking process, a series of complex reactions, including the Maillard reaction, lipid oxidation degradation, and thiamine degradation, will occur in flavor precursor substances in chicken meat. Volatile odors and flavor compounds produced during these reactions collectively constitute the overall flavor of chicken meat. In addition, lipid oxidation and the Maillard reaction are two important reactions that produce volatile compounds in chicken meat [46]. Aldehydes are key flavor compounds produced through the Strecker reaction of amino acids and the oxidation degradation of fatty acids [47,48]. These compounds are one of the main sources of meat flavor, and food containing aldehydes presents the scents of lipid, grass [49], and citrus. Compared to chicken meat in the C0 group, after the addition of CUR in chicken diets, chicken meat in different experimental groups all presented increased contents of aldehydes. Six aldehyde compounds, including hexaldehyde, heptaldehyde, octyl aldehyde, and n-nonyl aldehyde, exhibited a dosage effect (*p* < 0.05). Alcohols in the chicken meat primarily come from the oxidation degradation of unsaturated fatty acids. Alcohols can be divided into straight-chain alcohols and branched-chain alcohols. Straight-chain alcohols primarily come from lipid oxidation [46]. Branched-chain alcohols are mostly produced through the reduction of branched-chain aldehydes and the Strecker degradation of amino acids. These alcohols normally contain low-carbon chains. With the increase in the number of carbon chains, the flavor of alcohols changes from an anesthetic odor to a scent of fruit and lipids. From Table 5, it can be seen that a total of seven alcohol compounds were detected among the chicken meats in the C2 and C3 groups, and only six alcohol compounds were detected among the chicken meats in the C0 and C1 groups, with no 1-octen-3-ol detected.

Hydrocarbons can be divided into straight-chain hydrocarbons and olefins. Among them, straight-chain hydrocarbons have a relatively high threshold [50], thus exhibiting a relatively low contribution to meat flavor. After the addition of CUR in chicken diets, changes in the varieties and contents of hydrocarbons in chicken meat in different CUR-dosage groups were observed, with no trans-4,5-epoxydecane detected among the chicken meats in the C0 group. Myrcene presents a faint, meaty aroma. Chicken meat in the C3 group presented the highest contents of myrcene, which were significantly higher than those of chicken meat in the C0, C1, and C2 groups (*p* < 0.05). Ketone compounds mostly come from the oxidation degradation of fats. Most ketone compounds present aromas of milk and fruit. Ketones present a much higher threshold than that of aldehydes. Therefore, ketone compounds make a lower contribution to meat flavor than aldehyde compounds. Compared with chicken meat in the C0 group, after the addition of CUR in chicken diets, chicken meat presented a significantly increased content of ketones. Among all ketone compounds, chicken meat in different treatment groups presented the highest contents of 2,5-octanedione. Among all ester compounds that were detected, chicken meat in the C3 group presented the highest contents of ethyl caproate, which were significantly higher than those of chicken meat in all other groups (*p* < 0.05). Meanwhile, among all chicken meat samples, only two acid compounds, valeric acid, and caproic acid, were detected. Compared with chicken meat in the C0 group, chicken meat after the addition of CUR in chicken diets presented a significantly increased content of total acids (*p* < 0.05). Among all chicken meat samples, two heterocyclic compounds, benzoisothiazole and hydantoin, were detected. With an increased CUR addition amount in chicken diets, the contents of benzoisothiazole in chicken meat significantly increased (*p* < 0.05). Aromatic compounds that were detected in this study present a dosage effect on their total contents. Among these compounds, toluene and styrene hold a large proportion. Significant differences in the contents of aromatic compounds were observed among chicken meat samples in different groups, with the content of ortho-isopropyl toluene presenting a negative correlation with the CUR addition amount.

### 3.5. Identification of Key Aroma Components Based on OAV

Odor activity value (OAV) is an index used in the selection and identification of key flavor compounds in meat products. It is generally believed that an OAV greater than 1 indicates that the compound in question has a contribution to meat flavor. Meanwhile, a higher OAV indicates a higher contribution level of the compound to meat flavor [51]. From Table 6, it can be seen that a total of 13 flavor compounds with an OAV greater than 1 were detected among chicken meat samples in different treatment groups. Among them, there were 11 aldehyde compounds and 2 aromatic compounds. This indicates that all these 13 compounds make a significant contribution to the flavor of chicken meat. With a very low threshold, aldehydes exhibit a relatively high OAV and a significant contribution to meat flavor. Chicken meat in the CUR groups presents higher OAVs of hexaldehyde, heptaldehyde, octyl aldehyde, nonaldehyde, capraldehyde, and trans-2-decenal than those of chicken meat in the C0 group. Compared with chicken meat in all other groups, only chicken meat in the C2 group presents OAVs of myrcene greater than one, and only chicken meat in the C3 group exhibits OAVs of ethylbenzene greater than one. Benzaldehyde has a relatively low threshold and a high content in chicken meat. Therefore, its OAV is relatively high, making it a compound that contributes to the sweet flavor of chicken meat. Ethylbenzene is an aromatic compound that presents a unique aroma. Ethylbenzene itself presents a low contribution to the flavor of chicken meat. However, its concentrations in chicken meat continuously increases with the CUR addition amount in chicken diets, leading to the continuous increase in its contribution to meat flavor. Based on the above analysis, it can be concluded that the addition of CUR in chicken diets presents the most significant impact on the content of volatile aldehyde compounds in chicken meat. Compared with chicken meat in the C0 group, chicken meat in the CUR-dosage groups all presents increased OAVs of those 13 flavor compounds. This indicates that, with the addition of CUR in chicken diets, the flavor of chicken meat could be impacted through its affected content of volatile compounds.

## 4. Conclusions

The results show that the addition CUR in chicken feed is conducive to promoting the deposition of amino acids in chicken meat and improving its content of 5′-nucleotides, thus reducing the contents of SFA and C20:4 n6 and increasing the PUFA/SFA ratio in chicken meat. Moreover, after the addition of CUR in chicken diets, the volatile flavor characteristics of chicken breast meat can be improved. Particularly, its content of aldehydes can be significantly increased. Among them, daily feeding with 150 mg/kg CUR has the best effect on improving the nutritional value and flavor of chicken.

## Figures and Tables

**Table 1:** Ingredient composition and nutrient level of basal diets (dry-matter basis, %).

Daily Ration Composition %	1–21 Days of Age	22–43 Days of Age	Nutritional Level % ^2^	1–21 Days of Age	22–43 Days of Age
Corn	48.20	51.45	Metabolic energy/(MJ/kg)	12.76	13.39
Soymeal	41.80	36.50	Crude protein	24.14	22.91
Vegetable oil	5.00	7.05	Crude fiber	3.48	3.15
Premix compound ^1^	5.00	5.00	Calcium	0.83	0.81
Total	100.00	100.00	Total phosphorous	0.60	0.58
			Lysine	1.26	1.11
			Methionine	0.53	0.51

^1^ Premix compound provides the following substances for per kilogram of daily ration: VA, 10,000 IU; VD_3_, 4000 IU; VE, 20 IU; VK_3_, 2 mg; VB_1_, 2 mg; VB_2_, 6 mg; VB_6_, 3 mg; VB_12_, 0.02 mg; nicotinamide, 40 mg; calcium pantothenate, 10 mg; folic acid, 1 mg; biotin, 0.12 mg; Cu, 16 mg; Fe, 80 mg; Zn, 110 mg; Mn, 120 mg; I, 1.5 mg; Se, 0.3 mg. ^2^ Metabolic energy, crude protein, calcium, and total phosphorous values presented in the nutritional level item are all measured values, while all the other values are calculated values.

**Table 2:** Amino acid content of chicken breast meat samples (*n* = 8).

Amino Acid		Content (g/100 g)					
		C0 (0 mg/kg)	C1 (100 mg/kg)	C2 (150 mg/kg)	C3 (200 mg/kg)	SEM	*p*-Value
EAA	Thr	41.89 ^b^	26.93 ^d^	47.67 ^a^	39.15 ^c^	3.54	0.178
	Lys	29.98 ^b^	14.41 ^c^	34.77 ^a^	28.56 ^b^	2.89	0.012
	Val	35.02 ^b^	22.51 ^c^	41.12 ^a^	33.51 ^b^	3.15	0.187
	Met	23.47 ^b^	13.16 ^c^	26.63 ^a^	21.69 ^b^	2.05	0.05
	Ile	24.23 ^b^	6.04 ^d^	32.12 ^a^	17.91 ^c^	3.62	0.019
	Leu	3.10 ^b^	0.97 ^d^	3.98 ^a^	2.62 ^c^	0.42	0.018
	Phe	19.07 ^b^	8.50 ^c^	21.65 ^a^	17.87 ^b^	1.87	0.006
	Subtotal	176.76 ^b^	92.53 ^d^	207.93 ^a^	161.33 ^c^	16.79	0.034
NEAA	Asp	34.11 ^b^	23.91 ^c^	38.47 ^a^	34.01 ^b^	2.62	0.230
	Glu	45.01 ^b^	32.24 ^c^	49.90 ^a^	45.08 ^b^	3.19	0.218
	Ser	55.26 ^b^	29.60 ^d^	60.88 ^a^	33.91 ^c^	5.24	0.044
	Gly	22.11 ^a^	15.06 ^c^	17.07 ^b^	21.56 ^a^	2.27	0.681
	Ala	18.95 ^b^	11.46 ^c^	21.20 ^a^	18.11 ^b^	1.61	0.114
	Pro	22.45 ^b^	15.37 ^d^	26.50 ^a^	20.67 ^c^	2.01	0.283
	Cys	9.45 ^b^	5.00 ^c^	11.47 ^a^	9.64 ^b^	0.93	0.025
	His	14.89 ^b^	10.92 ^c^	16.40 ^a^	15.24 ^b^	1.19	0.417
	Arg	21.54 ^b^	13.13 ^d^	36.21 ^a^	19.36 ^c^	3.48	0.119
	Tyr	9.59 ^b^	3.07 ^c^	10.87 ^a^	9.01 ^b^	1.10	0.002
	Subtotal	253.37 ^b^	159.78 ^d^	288.99 ^a^	226.59 ^c^	20.68	0.112
TAA		430.13 ^b^	252.32 ^d^	496.93 ^a^	387.92 ^c^	37.27	0.068
SAA		200.09 ^b^	117.84 ^d^	219.57 ^a^	171.61 ^c^	16.80	0.104
BAA		141.32 ^b^	75.24 ^d^	178.11 ^a^	128.21 ^c^	14.45	0.035
FAA		79.13 ^b^	56.15 ^d^	88.38 ^a^	79.09 ^c^	5.81	0.223
EAA/TAA		41.09%	36.67%	41.84%	41.59%	0.00	0.034
EAA/NEAA		69.76%	57.91%	71.95%	71.20%	0.02	0.013

*n* = 8 replicates per treatment; different superscripts (a–d) means are significantly different in the same row. TAA = total amino acids; SAA = sweet amino acid (sum of Thr, Lys, Ser, Gly, Ala, and Pro); BAA = bitter amino acid (sum of Val, Met, Ile, Leu, Phe, Tyr, His, and Arg); FAA = flavor amino acid (sum of Asp and Glu).

**Table 3:** Nucleotide contents of chicken breast meat samples (*n* = 8).

Nucleotides	Content (g/100 g)					
	C0 (0 mg/kg)	C1 (100 mg/kg)	C2 (150 mg/kg)	C3 (200 mg/kg)	SEM	*p*-Value
GMP	0.38 ^d^	0.58 ^b^	0.86 ^a^	0.41 ^c^	0.07	<0.001
AMP	0.31 ^d^	0.77 ^a^	0.58 ^c^	0.67 ^b^	0.06	<0.001
IMP	0.05 ^d^	0.08 ^a^	0.07 ^b^	0.06 ^c^	0.00	<0.001

*n* = 8 replicates per treatment; different superscripts (a–d) means are significantly different on the same row.

**Table 4:** Fatty acid composition and content of chicken breast meat samples (*n* = 8).

Fatty Acid	Content (g/100 g)					
	C0 (0 mg/kg)	C1 (100 mg/kg)	C2 (150 mg/kg)	C3 (200 mg/kg)	SEM	*p*-Value
C10:0	0.01	0.01	0.01	0.01	0.00	<0.001
C12:0	0.02 ^c^	0.03 ^b^	0.02 ^c^	0.03 ^a^	0.00	<0.001
C13:0	0.01	0.01	0.01	0.01	0.00	<0.001
C14:0	0.32 ^c^	0.53 ^a^	0.29 ^d^	0.39 ^b^	0.03	<0.001
C15:0	0.10 ^b^	0.16 ^a^	0.10 ^b^	0.15 ^a^	0.10	<0.001
C16:0	6.55 ^b^	9.46 ^a^	6.09 ^c^	4.41 ^d^	0.89	0.261
C17:0	0.28 ^b^	0.35 ^a^	0.26 ^c^	0.34 ^a^	0.01	<0.001
C18:0	5.79 ^c^	7.57 ^a^	6.19 ^bc^	6.76 ^b^	0.26	0.005
C20:0	0.18 ^b^	0.20 ^a^	0.13 ^c^	0.20 ^a^	0.02	0.197
C21:0	0.03 ^b^	0.03 ^b^	0.07 ^a^	0.05 ^ab^	0.01	0.050
C22:0	0.09 ^b^	0.08 ^b^	0.12 ^a^	0.11 ^ab^	0.01	0.001
C23:0	0.10 ^a^	0.06 ^b^	0.09 ^a^	0.09 ^a^	0.01	0.171
C24:0	0.13 ^a^	0.05 ^a^	0.11 ^a^	0.10 ^a^	0.02	0.245
SFA	13.62 ^b^	18.52 ^a^	13.47 ^b^	12.64 ^c^	1.04	0.142
C14:1	0.10 ^c^	0.23 ^a^	0.09 ^c^	0.10 ^b^	0.02	<0.001
C16:1	3.28 ^b^	5.84 ^a^	2.98 ^c^	3.28 ^b^	0.44	<0.001
C18:1n9c	25.33 ^a^	15.13 ^b^	12.68 ^c^	24.12 ^a^	2.74	0.286
C20:1	0.08 ^b^	0.14 ^a^	0.08 ^b^	0.10 ^b^	0.01	0.013
C24:1	0.23 ^bc^	0.18 ^c^	0.50 ^a^	0.29 ^b^	0.05	0.001
MUFA	29.02 ^a^	21.51 ^b^	16.33 ^c^	27.90 ^a^	2.63	0.329
C18:2n6c	6.17 ^a^	0.44 ^d^	5.56 ^b^	3.86 ^c^	1.04	0.193
C18:3n6 (GLA)	0.38 ^c^	0.74 ^a^	0.34 ^c^	0.63 ^b^	0.06	<0.001
C18:3n3 (ALA)	3.86 ^b^	3.62 ^b^	3.07 ^c^	4.73 ^a^	0.23	0.001
C20:4n6 (AA)	5.98 ^c^	5.99 ^c^	12.16 ^a^	9.07 ^b^	1.02	0.018
C20:5n3 (EPA)	0.26 ^c^	0.44 ^b^	0.69 ^a^	0.43 ^b^	0.06	<0.001
C22:6n3 (DHA)	1.55 ^b^	1.43 ^b^	4.70 ^a^	2.03 ^b^	0.53	0.017
PUFA	18.21 ^c^	12.67 ^d^	26.52 ^a^	20.75 ^b^	2.06	0.048
PUFA/SFA	1.33 ^ab^	0.68 ^b^	1.97 ^a^	1.73 ^a^	0.21	0.069
n-3 PUFA	5.68 ^b^	5.50 ^b^	8.46 ^a^	7.19 ^ab^	0.49	0.031
n-6 PUFA	12.53 ^a^	7.17 ^d^	18.05 ^a^	13.56 ^c^	1.64	0.074

*n* = 8 replicates per treatment; different superscripts (a–d) means are significantly different on the same row. SFA—saturated fatty acid; PUFA—polyunsaturated fatty acid; PUFA/SFA—the ratio between contents of PUFAs and SFAs.

**Table 5:** Effects of dietary CUR on the volatile compounds of chicken breast meat samples (*n* = 8).

Volatile Compounds		Content (ng/g)					
		C0 (0 mg/kg)	C1 (100 mg/kg)	C2 (150 mg/kg)	C3 (200 mg/kg)	SEM	*p*-Value
Aldehydes	Hexanal	255.35 ^c^	716.89 ^b^	1514.93 ^a^	1637.96 ^a^	189.86	<0.001
	2-Hexenal,(E)-	0.25 ^c^	0.64 ^c^	3.07 ^a^	2.10 ^b^	0.37	<0.001
	Heptanal	9.57 ^c^	36.08 ^b^	45.74 ^b^	59.84 ^a^	6.27	<0.001
	2-Heptenal,(E)-	1.70 ^d^	4.84 ^c^	18.72 ^a^	14.23 ^b^	2.26	<0.001
	2,4-Heptadienal,(E,E)-	0.17 ^d^	0.68 ^c^	3.10 ^a^	2.13 ^b^	0.38	<0.001
	Octanal	6.89 ^c^	30.44 ^b^	38.76 ^b^	55.59 ^a^	5.94	<0.001
	2-methylundecanal	1.04 ^b^	2.17 ^a^	1.80 ^a^	1.81 ^a^	0.17	0.022
	2-Octenal,(E)-	0.90 ^d^	3.61 ^c^	14.22 ^a^	11.00 ^b^	1.77	<0.001
	Nonanal	8.84 ^d^	43.94 ^c^	62.78 ^b^	97.87 ^a^	10.70	<0.001
	1,3,4-trimethyl-3-Cyclohexene-1-carboxaldehyde,	1.32 ^b^	2.2 ^a^	0.09 ^c^	0.74 ^b^	0.27	0.002
	2-Nonenal,(E)-	0.15 ^c^	0.69 ^b^	2.28 ^a^	2.25 ^a^	0.31	<0.001
	Decanal	0.39 ^d^	1.53 ^c^	2.69 ^b^	3.98 ^a^	0.44	<0.001
	2,4-Nonadienal,(E,E)-	0.40 ^d^	1.88 ^c^	8.38 ^a^	7.01 ^b^	1.10	<0.001
	2-Decenal, (E)-	0.18 ^c^	0.95 ^b^	3.87 ^a^	4.03 ^a^	0.56	<0.001
	2,4-Decadienal,(E,Z)-	0.15 ^d^	0.72 ^c^	2.98 ^a^	2.11 ^b^	0.37	<0.001
	Undecanal	0.07 ^d^	0.67 ^a^	0.35 ^b^	0.21 ^c^	0.11	0.146
	2,4-Decadienal,(E,E)-	0.32 ^d^	1.75 ^c^	7.00 ^a^	5.07 ^b^	0.88	<0.001
	2-Undecenal,(E)-	0.09 ^c^	0.38 ^b^	1.61 ^a^	1.58 ^a^	0.23	<0.001
	Dodecanal	0.10 ^d^	0.39 ^c^	1.32 ^a^	0.90 ^b^	0.16	<0.001
	Tridecanal	-	0.45	0.84	0.64	0.12	0.018
	Pentadecanal	0.18 ^c^	0.45 ^b^	0.9 ^a^	0.69 ^ab^	0.09	0.002
Subtotal		288.05 ^c^	851.36 ^b^	1735.39 ^a^	1911.69 ^a^	219.85	<0.001
Hydrocarbons	1,2-epoxyheptane	1.34 ^c^	5.03 ^b^	6.27 ^a^	7.12 ^a^	0.76	<0.001
	Bornyl bromide	1.29 ^a^	2.00 ^a^	0.09 ^b^	0.12 ^b^	0.28	0.002
	4,5-Epoxynonane,(E)-	-	0.09 ^b^	0.27 ^a^	0.12 ^b^	0.04	0.076
	Tetradecane	0.29 ^b^	0.75 ^a^	0.80 ^a^	0.94 ^a^	0.09	0.013
	Heneicosane	0.73 ^b^	1.62 ^ab^	2.21 ^a^	2.19 ^a^	0.24	0.056
	Pentadecane	0.27 ^b^	0.57 ^b^	1.34 ^a^	1.37 ^a^	0.18	<0.001
	n-Hexadecane	0.40 ^d^	0.66 ^c^	0.99 ^a^	0.87 ^b^	0.11	0.017
	1,3-Octadiene	0.42 ^c^	1.57 ^b^	3.40 ^a^	3.24 ^a^	0.41	0.250
	β-myrcene	25.02 ^c^	24.65 ^c^	29.01 ^b^	33.15 ^a^	1.85	0.408
	2,6-Dimethyl-1,3,5,7-octatetrene,(3 E,5 E)-	1.96 ^b^	3.22 ^a^	0.13 ^c^	0.18 ^c^	0.44	<0.001
	2-Carene	1.39 ^a^	1.55 ^a^	0.03 ^b^	0.07 ^b^	0.26	0.008
	1,3-Hexadiene,3-ethyl-2-methyl-	275.71 ^b^	689.55 ^a^	17.46 ^c^	13.23 ^d^	101.38	0.008
	D-Limonene	0.61 ^d^	0.93 ^c^	3.99 ^a^	3.60 ^b^	0.50	<0.001
	beta.-Ocimene	2.83 ^a^	2.84 ^a^	0.08 ^b^	0.17 ^b^	0.49	0.010
	.gamma.-Terpinene	0.93 ^c^	1.25 ^b^	1.601 ^a^	1.25 ^b^	0.11	0.224
	Cyclohexene,1-methyl-4-(1-methylethylidene)-	1.06 ^b^	1.65 ^a^	0.13 ^c^	0.16 ^c^	0.23	0.010
	Limonene oxide,(E)-	2.42 ^b^	5.69 ^a^	0.02 ^d^	1.04 ^c^	0.74	<0.001
	3-Octadecene	0.8 ^c^	1.62 ^b^	2.21 ^a^	2.19 ^a^	0.21	0.006
	(E)-9-Octadecene	0.13 ^d^	0.35 ^c^	0.75 ^a^	0.50 ^b^	0.09	0.044
	Butanoic acid,anhydride	0.23 ^b^	1.16 ^a^	1.06 ^a^	0.91 ^a^	0.13	<0.001
Subtotal		317.82 ^b^	746.75 ^a^	71.79 ^c^	72.35 ^c^	101.11	0.007
Ketones	Hexanone,4-hydroxy-3-propyl-	0.58 ^d^	1.49 ^c^	2.03 ^b^	1.80 ^a^	0.21	0.023
	3-Heptanone	0.39 ^c^	0.75 ^b^	1.46 ^a^	1.45 ^a^	0.16	<0.001
	2-Heptanone	9.21 ^c^	33.37 ^b^	57.31 ^a^	63.55 ^a^	7.28	<0.001
	Heptanone,6-methyl-	0.90 ^d^	6.19 ^c^	10.52 ^a^	8.49 ^b^	1.22	<0.001
	3-Ethylcyclopentanone	0.50 ^c^	1.87 ^b^	3.95 ^a^	3.31 ^a^	0.45	<0.001
	2,5-Octanedione	37.27 ^d^	173.13 ^c^	254.94 ^b^	288.56 ^a^	32.98	<0.001
	3,5-Octadien-2-one	0.18 ^c^	1.03 ^b^	2.63 ^a^	2.84 ^a^	0.37	<0.001
	2-Nonanone	0.96 ^c^	1.09 ^bc^	1.15 ^b^	1.36 ^a^	0.06	0.087
	Cyclohexanone,2-methyl-5-(1-methylethenyl)-,trans-	3.32 ^b^	5.80 ^a^	0.91 ^d^	1.05 ^c^	0.68	<0.001
	2-Undecanone	0.24 ^c^	1.03 ^b^	1.61 ^a^	1.58 ^a^	0.21	0.012
	trans-3-Nonen-2-one	0.18 ^d^	1.16 ^c^	6.92 ^a^	5.85 ^b^	0.96	<0.001
	11-Heneicosanone	-	-	0.01 ^b^	0.16 ^a^	0.02	0.006
Subtotal		53.72 ^c^	226.92 ^b^	343.42	379.96 ^a^	43.22	<0.001
Alcohols	3,5-Octadien-2-ol	0.11 ^d^	1.92 ^c^	3.79 ^a^	3.43 ^b^	0.49	<0.001
	Benzyllinalool	1.24 ^b^	1.25 ^b^	2.3 ^a^	2.48 ^a^	0.21	0.010
	1,3-Dioxolane-2-butanol,2-methyl-	0.10 ^d^	0.70 ^c^	3.89 ^b^	5.04 ^a^	0.68	<0.001
	2,5-Dimethylcyclohexanol	0.31 ^d^	2.18 ^c^	11.85 ^a^	10.04 ^b^	1.62	<0.001
	Linalool	0.04 ^c^	0.06 ^bc^	0.11 ^a^	0.07 ^b^	0.01	<0.001
	2-Octen-1-ol,(E)-	0.05 ^d^	0.42 ^c^	1.75 ^a^	0.64 ^b^	0.20	<0.001
	1-Octen-3-ol	-	-	0.64 ^a^	0.03 ^b^	0.01	<0.001
Subtotal		1.85 ^d^	6.53 ^c^	23.73 ^a^	21.71 ^b^	3.10	<0.001
Esters	Caproic acid vinyl ester	0.13 ^d^	1.02 ^c^	4.3 ^b^	5.97 ^a^	0.78	<0.001
	Butyrolactone	0.2 ^d^	0.82 ^a^	0.46 ^c^	0.66 ^b^	0.09	0.011
	Linalyl acetate	0.54 ^b^	0.88 ^a^	0.12 ^c^	0.08 ^c^	0.12	0.003
	Carbamodithioic acid,diethyl-,methyl ester	6.49 ^b^	9.14 ^a^	1.16 ^c^	0.49 ^d^	1.23	<0.001
Subtotal		7.35 ^b^	11.86 ^a^	6.02 ^b^	7.19 ^b^	0.86	0.016
Aromatic	Toluene	8.82 ^d^	11.04 ^c^	33.46 ^b^	68.51 ^a^	7.74	<0.001
	Ethylbenzene	2.10 ^c^	2.73 ^c^	14.84 ^b^	32.68 ^a^	3.99	<0.001
	p-Xylene	5.76 ^a^	6.41 ^a^	1.95 ^b^	6.93 ^a^	0.66	0.011
	Styrene	1.05 ^c^	1.13 ^c^	46.29 ^b^	113.95 ^a^	14.82	<0.001
	Benzene,1,3-dimethyl-	2.87 ^c^	3.82 ^bc^	4.32 ^b^	6.93 ^a^	0.50	0.002
	Benzene,(1-methylethyl)-	0.05 ^c^	0.1 ^c^	1.93 ^b^	4.49 ^a^	0.58	<0.001
	Benzaldehyde	11.90 ^c^	15.02 ^bc^	21.92 ^a^	18.39 ^ab^	1.35	0.011
	o-Cymene	37.90 ^b^	54.47 ^a^	0.35 ^c^	0.30 ^c^	8.09	0.001
	Benzeneacetaldehyde	1.20 ^b^	1.83 ^ab^	2.30 ^a^	2.48 ^a^	0.20	0.044
	Benzaldehyde, 4-ethyl-	0.20 ^c^	1.52 ^b^	3.51 ^a^	3.77 ^a^	0.49	<0.001
Subtotal		71.84 ^d^	98.08 ^c^	130.84 ^b^	258.4 ^a^	23.16	0.000
Acids	Pentanoic acid	0.29 ^c^	1.18 ^b^	2.08 ^a^	2.34 ^a^	0.29	0.007
	Hexanoic acid	2.54 ^d^	15.02 ^c^	38.86 ^b^	51.99 ^a^	6.47	<0.001
Subtotal		2.83 ^d^	16.20 ^c^	40.94 ^b^	54.33 ^a^	6.75	0.000
Heterocyclic	1,2-Benzisothiazole	0.28 ^d^	0.40 ^c^	7.04 ^b^	11.21 ^a^	1.50	<0.001
	Hydantoin	0.13 ^c^	1.50 ^a^	0.21 ^b^	0.12 ^c^	0.21	<0.001
Subtotal		0.40 ^d^	1.90 ^c^	7.24 ^b^	11.33 ^a^	1.42	0.000

*n* = 8 replicates per treatment; different superscripts (a–d) means are significantly different on the same row.

**Table 6:** The OAV of volatile flavor compounds in breast muscles of chicken (*n* = 8).

Compound	Odor Threshold (μg/kg)	Odor Description	OAV			
			C0 (0 mg/kg)	C1 (100 mg/kg)	C2 (150 mg/kg)	C3 (200 mg/kg)
Hexanal	5	Green, grassy, fat	51.07	143.38	302.99	327.59
Hexanal	3	Fresh, burnt fat	3.19	12.03	15.25	19.95
Octanal	1	Fatty, green	6.89	30.44	38.76	55.59
Trans-2-octenal	0.08	fruit, Fatty	11.28	45.11	177.67	137.50
Nonanal	1	Fatty, green	8.84	43.94	62.78	97.87
Trans-2-nonenal	0.08	Fatty	1.88	8.56	28.49	28.15
Decanal	0.1	Green, onion, yeast	3.90	15.33	26.91	39.75
2,4-nonadienal,(E,E)-	0.06	Flower, Fatty	6.61	31.29	139.55	116.84
Trans-2-decenal	0.3	Sweet orange	0.59	3.19	12.89	13.42
-2,4-Decadienal,(E,E)	0.7	Fatty, toasted, scallion	0.45	2.50	10.00	7.24
Lauric aldehyde	0.9	Flower	0.11	0.44	1.46	0.99
Benzaldehyde	3	Nutty, bitter almond, burnt sugar	3.96	5.01	7.31	6.13
Ethyl benzene	29	Fragrant	0.07	0.09	0.51	1.13

## Data Availability

The original contributions presented in the study are included in the article, further inquiries can be directed to the corresponding author.
